# Comparison of Muscle Activity between the Horizontal Bench Press and the Seated Chest Press Exercises Using Several Grips

**DOI:** 10.5114/jhk/161468

**Published:** 2023-04-20

**Authors:** José M. Muyor, David Rodríguez-Ridao, José M. Oliva-Lozano

**Affiliations:** 1 Health Research Centre, Faculty of Educational Sciences, University of Almería, Almería, Spain.

**Keywords:** electromyography, muscle excitation, muscle activation, fitness, strength

## Abstract

This study aims to compare muscle activity in the pectoralis major, anterior deltoid, and triceps brachii in the horizontal bench press exercise with a prone grip at 150% and 50% of the biacromial width and the seated chest press exercise with two types of grips (a neutral grip at ~150% of the biacromial width and a prone grip at ~200% of the biacromial width). Twenty physically active adults performed a set of 8 repetitions at 60% of the one repetition maximum. The results showed that the clavicular portion of the pectoralis major had significantly greater muscle activity in the seated chest press exercise with a neutral grip (~30% of the maximal voluntary isometric contraction (MVIC)) than in the lying bench press exercise with a prone grip at 150% of the biacromial width (~25% MVIC). The muscle activity of the anterior deltoid was not significantly different across any exercise or grip evaluated (~24% MVIC). The muscle activity of the triceps brachii was significantly higher in the lying bench press exercise with a grip at 50% biacromial width (~16% MVIC) than at 150% of the biacromial width (~12% MVIC). In conclusion, all exercises and grips showed similar muscle activity, and the selection of these exercises should not be based exclusively on the grounds of muscle activation but rather on the load capacity lifted, the level of technique of the participant, and/or the transference to the specific sporting discipline or event.

## Introduction

Currently, it is well known that strength training has positive effects on sports performance and the health of individuals (Pichardo et al., 2019; Westcott, 2012). For strength training and strength and conditioning, professional trainers usually propose two types of exercises: those performed on guided machines, usually for beginners or with little command of technical execution, and those performed with free weights for athletes with more advanced execution techniques (Baechle and Groves, 1998).

Therefore, it is essential to correctly select the exercises that generate the necessary adaptations in the appropriate muscles. One of the most commonly used techniques to determine the degree of muscle activation and identify activation patterns of several muscles is surface electromyography (sEMG) (David et al., 2000; Strońska et al., 2018).

sEMG measures the electrical potential present on the skin due to muscle contraction (Merlo and Campanini, 2010), and it is highly correlated with muscle force (Disselhorst-Klug et al., 2009).

The bench press exercise is a popular and widely used example of this kind of exercise that allows upper-limb muscle strengthening through additional loads (Giorgio et al., 2009). It is a complex exercise in which large external loads can be lifted, requiring high neuromuscular activity (Stastny et al., 2017). In this regard, the bench press exercise is often modified by bench inclination (Rodríguez-Ridao et al., 2020; Saeterbakken et al., 2017), grip width (Barnett et al., 1995; Lehman, 2005; Saeterbakken et al., 2017), or using a Sling shot (Wojdala et al., 2022) to adjust the muscle activity of the primary movements.

A previous study by Barnett et al. (1995) addressed the influence of grip width and bench inclination on the muscle activity of the primary movements during the bench press exercise with two grip widths at 100% and 200% of the biacromial distance (a narrow and a wide grip, respectively). Those authors found that the horizontal bench press exercise with a narrow grip produced a higher sEMG activity of the clavicular head of the pectoralis major and the anterior deltoid than with a wide grip. With the trunk in a vertical position, hand spacing had no effect on the sEMG activity in the evaluated muscles. However, those authors evaluated the bench press exercise with the participants seated on a bench and with the trunk vertical. Consequently, the load was lifted vertically, as in a military press exercise, and not pushed in a horizontal plane. Alternatively, Lehman (2005) evaluated the influence of three grip widths (100% and 200% of the biacromial distance and a pronated grip with hand width distance between the two hands) during the flat bench press exercise on the muscle activity of the clavicular head of the pectoralis major, the lateral head of the triceps, and the biceps brachii. That author reported that changing from the widest to the narrowest grip width increased the sEMG activity in the triceps brachii, independent of the hand position (prone or supine) and decreased the sEMG activity of the sternoclavicular portion of the pectoralis major with a prone grip. However, these authors did not assess the muscle activity in the lower portion of the pectoralis major or the anterior deltoid muscle. In this regard, the anterior deltoid is an essential muscle in shoulder flexion, and the pectoralis major, the lower portion, has an indispensable role in the pushing action. Therefore, electromyographic evaluation of these muscles could provide more information on the involvement of these muscles in the bench press and seated chest press exercises with their grip variations. Moreover, some authors have reported that when, in the bench press exercise, the hand spacing is > 200% of the biacromial distance, the shoulder position puts the athlete at a high risk of injury (Gross et al., 1993); therefore, the grip should not exceed 150% of the biacromial distance (Green and Comfort, 2007). Saeterbakken et al. (2017) compared the sEMG activity in the bench press exercise in the style of competition with either the +25° inclined and −25° declined bench position (wide grip) or using a narrow and medium grip (flat bench). These authors concluded that there were no differences in sEMG activity during the wide grip and on a flat bench in chest and shoulder muscles compared with inclined and declined bench positions and in medium and narrow grip width. However, the biceps brachii showed greater and the triceps brachii lower sEMG activity during the inclined bench press than the flat and declined bench position.

Recently, Rodríguez-Ridao et al. (2020) evaluated the muscle activity of the pectoralis major, anterior deltoid, and triceps brachii during the lying bench press exercise in five bench inclinations, using only one grip width at 150% of the biacromial distance. Those authors found that as the bench inclination increased, the sEMG activity of the anterior deltoid also increased. Additionally, they reported that the upper portion of the pectoralis major (PMUP) showed the highest sEMG activity at 30° of bench inclination. However, they did not analyse the sEMG activity regarding grip width.

Alternatives to the bench press exercise are guided weight machines, which are considered safe, effective, and easy to learn and are also an alternative to free weights (American College of Sports Medicine, 2009). However, the presence of machine constraints reducing the degree of freedom in multijoint exercises can conceivably influence muscle activity patterns by altering the muscle forces required for movement execution and stabilization, affecting the specificity and effectiveness of strength training (Cacchio et al., 2008).

Several studies have compared the muscle activation of the bench press exercise to other exercises performed in guided machines, such as the Smith machine (Saeterbakken et al., 2011; Schick et al., 2010) or the peck deck (de Araújo Rocha Júnior et al., 2007), in relation to other exercises such as the pullover (Campos and da Silva, 2014), the standing cable press (Santana et al., 2007; Signorile et al., 2017) or with free weights (Saeterbakken et al., 2011). However, few studies have analysed whether there are differences in muscle activation during the bench press exercise compared to the seated chest press machine and its grip derivates. Wattanaprakornkul et al. (2011) compared shoulder muscle activity to the seated chest press machine and a specific shoulder flexion lying prone on a bench. However, those authors did not evaluate muscle activity during the bench press exercise. Recently, Coratella et al. (2020) found that the sternocostal head of the pectoralis major and the anterior deltoid were similarly activated, and the triceps brachii and the lateral deltoid were primarily recruited in the bench press exercise rather than in the seated chest press machine. However, those authors only evaluated the seated chest press with a prone grip.

Therefore, this study aimed to: 1) compare muscle activity, through sEMG, in the clavicular portion, sternal portion, and costal portion of the pectoralis major, anterior deltoid, and the long head of the triceps brachii during the bench press exercise with two grip widths (at 150% and 50% of the biacromial width), and the seated chest press exercise with both types of grips (prone and neutral); and 2) analyse which muscles would have the greatest sEMG activity in each of these evaluated exercises.

The primary hypothesis was that the muscle activity in all analysed muscles would be similar between the lying bench press exercise with a grip at 150% of the biacromial width and the seated chest press exercise, independent of the grip type (prone or neutral). However, we hypothesized that the bench press exercise with a grip at 50% of the biacromial width would show greater muscle activity in the triceps brachii compared to the same exercise with a broader grip width.

## Methods

### Participants

A total of 20 physically active adults (age: 22.90 ± 2.98 years, body mass: 75.00 ± 8.75 kg, body height: 1.77 ± 0.04 m, body mass index: 23.96 ± 2.12) with 5.03 ± 1.01 years of experience in strength training participated in this study.

To be included in the study, participants were required to meet the following inclusion criteria: 1) have a minimum experience of 4 years of strength training, with a weekly frequency of at least twice a week of resistance training; 2) no injury or limitation that would impact the performance of the exercises proposed in the study; 3) no history of upper or lower spinal surgery; and 4) no use of any drugs, anabolic agents or drinks that could alter the study results. Moreover, all participants were asked to avoid any vigorous exercise and not to ingest stimulant drinks 24 hours before the measurements.

The sample size was a priori calculated using G*Power software (version 3.1) for Mac OSX (Faul et al., 2007), with a statistical power (1 – ß) of 0.8, a significance level of 0.05, and an effect size of 0.6. A final sample size of 19 subjects was obtained, although in the current study, a sample size of 20 participants was used.

Prior to enrolling in the study, all participants received verbal and written information on the purposes and methods of the study, which had been developed in accordance with the Declaration of Helsinki and authorized by the Bioethical Committee of the University of Almería. An informed consent form was freely signed by each participant.

### Procedures

Each participant went to the laboratory on two occasions with at least 48 hours between each visit to avoid muscle fatigue. During the first visit, participants reached the maximal lifting load in a unique repetition, i.e., one repetition maximum (1RM) of the bench press exercise gripping the bar (using a hook grip with the thumb) at 150% and 50% of the biacromial width and the seated chest press exercise with both a prone and a neutral grip. Participants performed the lifts in random order for each exercise to minimize any possible order effect. The second visit was conducted to evaluate the muscle activity in each exercise.

### Determination of the 1RM

Following the protocol described by Rodríguez-Ridao et al. (2020), the first session began by evaluating each participant’s biacromial width, body mass (measured with an electronic body composition analyser (model BF−350; Tanita, Tokyo, Japan)), and height (measured with a Seca stadiometer (Seca, Hamburg, Germany)).

Afterwards, participants warmed up on an elliptical machine. Then, they performed joint mobility and active stretching exercises of the upper limbs for 3–5 minutes. Next, participants rested for 3–5 minutes to avoid possible fatigue before the tests.

Participants then performed a specific protocol to reach the 1RM in the bench press exercise with both grip widths (at 150% and 50% of the biacromial width) and in the seated chest press exercise with both types of grips (prone and neutral). This protocol by Saeterbakken et al. (2011) was as follows: 1) 20 repetitions at approximately 30% 1RM, 2) 12 repetitions at approximately 50% 1RM, 3) six repetitions at approximately 70% 1RM, and 4) one repetition at approximately 85% 1RM.

Last, participants had to lift the most weight they could manage in one repetition (1RM) while using the appropriate form (Kraemer and Fry, 1995). Through this method, 1RM was usually established in the second attempt. However, when necessary, a third attempt was made. The rest intervals between sets and between the bench press and seated chest press exercises were approximately five minutes long to avoid muscle fatigue. None of the displayed signs of weariness would have interfered with the accuracy or validity of the tests. The 1RM for each exercise and the grip variations were calculated in the morning and later randomized and counterbalanced. The results of the 1RM are shown in Table 1.

**Table 1:** Mean ± standard deviation 1RM for the four exercises evaluated.

	Mean ± SD
1RM Lying barbell bench press (kg) with a grip at 150% of the biacromial width (A)	85.00 ± 12.87
1RM Lying barbell bench press (kg) with a grip at 50% of the biacromial width (B)	69.25 ± 14.80
1RM Seated machine chest press with a prone grip (kg) (C)	79.25 ± 14.98
1RM Seated machine chest press with a neutral grip (kg) (D)	69.75 ± 11.05

Significant differences in 1RM between exercises: p < 0.001: A vs. B; A vs. D; C vs. D p < 0.01: A vs. C; B vs. C

### Electromyography Setup and Data Collection

This protocol was performed at the second visit, which started with the same warm-up as in the first visit. Corporal areas were then chosen for electrode placement and were prepared by shaving the hair and cleansing the area with alcohol to reduce surface impedance. Ag/AgCl electrodes (Medico Lead-Lok, Noida, India) were placed parallel to the muscle fibres at a 2 cm centre-to-centre distance.

To avoid any potential displacement during the activities, the electrodes were then placed on each participant's dominant side and secured using adhesive tape. Particularly, the electrodes were positioned in accordance with the suggestions made by Surface Electromyography for the Non-invasive Assessment of Muscles (SENIAM) (Hermens et al., 2000), on muscles following a detailed description (Table 2).

**Table 2:** Surface electrode placement and maximal isometric voluntary contraction (MVIC) manoeuvre description

Muscle	Electrode placement	MVIC manoeuvre
Pectoralis major upper portion (clavicular portion, PMUP)	On the midclavicular line over the second intercostal space (Glass and Armstrong, 1997).	In a standing position, with shoulders and elbows flexed at 90° (in the horizontal plane), the participants brought their elbows towards their body’s midline (simulating the pec-deck exercise) against maximal manual resistance in the opposite direction.
Pectoralis major middle portion (sternal portion, PMMP)	On the chest wall horizontally from the arising muscle mass (approximately 2 cm out from the axillary fold) (Park et al., 2013).	
Pectoralis major lower portion (costal portion, PMLP)	At the midclavicular line over the fifth intercostal space (Glass and Armstrong, 1997).	
Anterior deltoid (AD)	At 1.5 cm distal and anterior to the acromion (Saeterbakken and Fimland, 2013).	Participants performed a deltoid flexion at 90° in a seated position with an erect posture and no back support against maximal manual resistance in the opposite direction.
Triceps brachii (TB) long head	At the midpoint between the posterior aspect of the acromion and the olecranon processes (Cogley et al., 2005).	Participants performed a forearm extension with elbows at 90° in a seated position with an erect posture and no back support against maximal manual resistance in the opposite direction.

To standardize the sEMG readings recorded during the bench press and seated chest press exercises, the maximum voluntary isometric contraction (MVIC) of each muscle was recorded after the electrodes were placed. To this end, two 3-s MVICs trials were recorded for each muscle in a randomized manner, with approximately a 10-s rest interval between each contraction and a 2-min rest interval between the MVIC measurement of each muscle (Muyor et al., 2019). The MVIC was determined as an average amplitude over a one- second window of the highest rectified sEMG signals (root-mean-square, RMS) with a 100 ms window (Contreras et al., 2016). The intraclass correlation coefficient (ICC) values were calculated to verify the consistency between repetitions for the MVIC tests. Additionally, the percentage of the coefficient of variation (CV) was calculated. The ICCs were > 0.95 (0.94–0.98; *p* < 0.000) and CV was < 3% in the sEMG in all MVIC assessments.

The MVIC manoeuvres are shown in Table 2. All muscles were randomly tested to avoid fatigue. Additionally, each participant received verbal support from an examiner to maintain a continuous effort throughout the MVIC.

Accordingly, to start the test each participant completed a more focused warm-up that included 15 repetitions at 30% of the exercise. Last, after a 5-min rest interval, the bench press and seated chest press exercises data were collected for each grip condition in random and counterbalanced order with a 5-min rest interval between trials. The sEMG signal was registered while participants performed a set of 8 repetitions at 60% 1RM (Rodríguez-Ridao et al., 2020), and the eccentric phase and the concentric phase were performed and recorded at a velocity of two seconds in each phase (Wattanaprakornkul et al., 2011) for each exercise and grip condition. This velocity was controlled by a KORG MA−1 metronome (Keio Electronic Laboratories, Tokyo, Japan) (Muyor et al., 2019; Rodríguez-Ridao et al., 2020).

**Figure 1:**
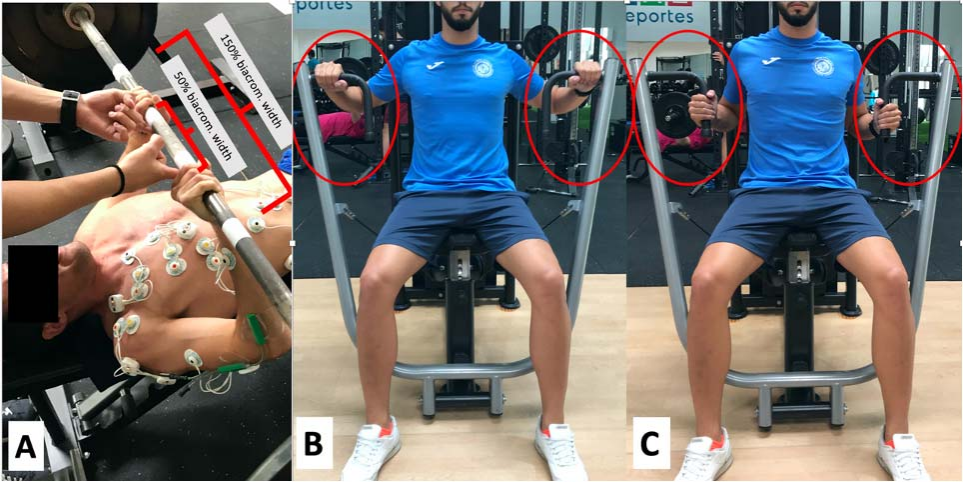
Horizontal bench press exercise with a prone grip at 150% and 50% of the biacromial width (A). Seated chest press exercise with a prone grip (B) and a neutral grip (C).

Regarding technique, all participants began the bench press exercise supine on the bench, gripping the bar with their thumbs in a hook grip at either 150% or 50% of the biacromial width, depending on the type of bench press exercise they were performing. Participants also kept their hands and forearms pronated throughout all repetitions (Figure 1A). To maintain the starting position, the bar was lowered to 1 cm from the chest (sternum), the shoulders were abducted to approximately 45° (during the eccentric phase), and the bar was raised until the elbows were extended (Muyor et al., 2019). Regarding the technique during the seated chest press exercise, all participants started the exercise seated on the machine, gripping the bar with their thumbs in a hook grip and the hands and forearms pronated (a prone grip) (Figure 1B) or in a prone-supine position (a neutral grip) (Figure 1C) during all repetitions recorded.

### Electromyography

Using a WBA Mega device (Mega Electronics, Ltd., Kuopio, Finland), sEMG data for each muscle were captured and sampled at 1000 Hz. An A/D converter (National Instruments, New South Wales, Australia) was used to convert the analogue signal to a digital signal. LabView software (National Instruments, Austin, TX, USA) was then used to filter the digital signal by bandwidth (12–450 Hz) using a fourth-order Butterworth filter. The MEGAWIN software program (Mega Electronics, Ltd.) was used to transform the raw sEMG data into RMS signals in microvolts (V) for further analysis.

Although data were collected during eight repetitions, the first (initial) and the last (8^th^) were discarded to eliminate movement variability due to initiation and termination of the exercise (Cacchio et al., 2008).

### Statistical Analyses

The Shapiro-Wilk normality test was used to analyse the data distribution. Parametric tests were performed because all variables followed a normal distribution.

Two separate repeated-measures ANOVAs were performed to analyse the dependent variable of muscle activity. A 4 x 5 ANOVA (exercise*muscle) design was applied to determine differences in each muscle activity (% MVIC) across exercises and their variants of grips. Likewise, a 5 x 4 ANOVA (muscle*exercise) design was applied to determine differences in the muscle activity (% MVIC) across different muscles in each exercise and their variants of grips. Additionally, to assess assumptions of variance, the Mauchly's test of sphericity was performed using all the ANOVA results. A Greenhouse-Geisser correction was performed to adjust the degrees of freedom if an assumption was violated. When significant *F* values were obtained, pairwise comparisons using a Bonferroni adjustment were employed. Partial eta-squared (η^2^_p_) was used to estimate explained variance and effect size, and a value of 0.2 was considered a small effect, 0.5 a medium effect, 0.8 a large effect, and 1.3 a very large effect (Levine and Hullett, 2002).

Statistical analyses were carried out using IBM SPSS software (v.27), and the level of significance was set at *p* < 0.05.

## Results

**Figure 2:**
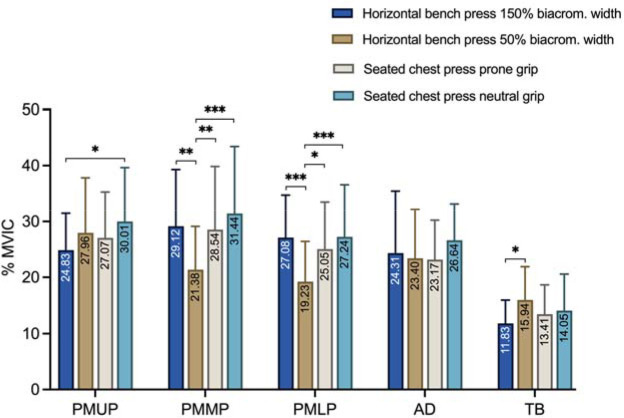
Comparison of the electromyographic activity of each muscle between exercises and grips. PMUP: pectoralis major upper portion (clavicular portion); PMMP: pectoralis major middle portion (sternal portion); PMLP: pectoralis major lower portion (costal portion); AD: anterior deltoid; TB: triceps brachii (long head). * p < 0.05; ** p < 0.01; *** p < 0.001

ANOVA indicated that the main effect of exercise on muscle activity for the PMUP was statistically significant with a small effect size (F_(3,57)_ = 4.33, *p* = 0.008, η^2^_p_ = 0.18), with post hoc testing indicating that the muscle activity for the PMUP in the seated chest press exercise was significantly greater than in the lying bench press exercise (*p* = 0.016). The main effect of exercise on muscle activity for the middle portion of the pectoralis major (PMMP) was statistically significant with a small effect size (F_(3,57)_ = 11.79, *p* < 0.001, η^2^_p_ = 0.38) with post hoc testing indicating that the muscle activity for the PMMP in the lying bench press exercise with a grip at 50% of the biacromial width was significantly lower than the rest of the exercises (*p* ≤ 0.01). The main effect of exercise on muscle activity for the anterior deltoid (AD) was not statistically significant. Moreover, it showed a very small effect size (F_(3,57)_ = 1.20, *p* = 0.318, η^2^_p_ = 0.05), with post hoc testing indicating that the muscle activity for the AD was not significantly different between any exercise (*p* > 0.05). The main effect of exercise on muscle activity for the triceps brachii (TB) was statistically significant with a small effect size (F_(1.63, 30.96)_ = 3.90, *p* < 0.038, η^2^_p_ = 0.17), with post hoc testing indicating that the muscle activity for the TB in the lying bench press exercise with a grip at 50% of the biacromial width was significantly greater than that in the same exercise with a grip at 150% of the biacromial width (*p* = 0.025). The specific values and their comparisons are detailed in Figure 2.

**Figure 3:**
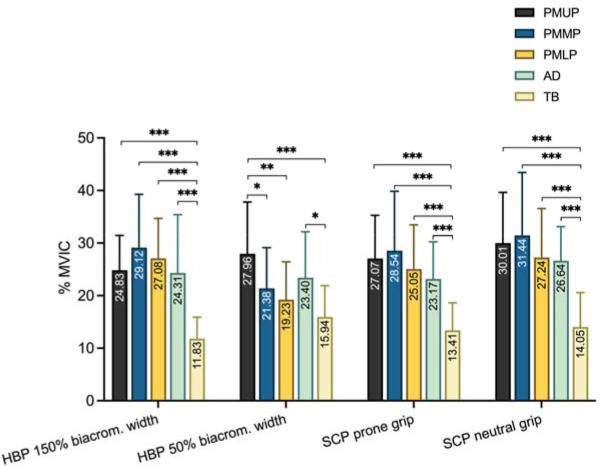
Comparison of electromyographic activity between muscles in each exercise and grip. HBP: horizontal bench press; SCP: seated chest press; PMUP: pectoralis major upper portion (clavicular portion); PMMP: pectoralis major middle portion (sternal portion); PMLP: pectoralis major lower portion (costal portion); AD: anterior deltoid; TB: triceps brachii (long head). * p < 0.05; ** p < 0.01; *** p < 0.001

Regarding the muscle activity in each exercise (Figure 3), the PMMP showed the highest muscle activity in all exercises, except in the lying bench press exercise with a grip at 50% of the biacromial width. There were no significant differences in the muscle activity between the upper, middle, and lower portions of the pectoralis major and the AD in any exercise evaluated, except for the lying bench press exercise with a grip at 50% of the biacromial width, where the PMUP had significantly greater muscle activity than the PMMP and the lower portion of the pectoralis major (PMLP) (Figure 3).

## Discussion

Coaches often prescribe different strength exercises and variations in an attempt to activate or modify the activation of the musculature involved in the primary movements. In this regard, one of the main aims of the current study was to compare muscle activity in the pectoralis major (clavicular portion, sternal portion, and costal portion), anterior deltoid, and the long head of the triceps brachii in the bench press exercise with two grip widths (at 150% and 50% of the biacromial width) and in the seated chest press exercise with two types of grips (prone and neutral). When comparing the muscle activity of each muscle among the different exercises and grips, the main result was that the PMUP showed significantly greater muscle activity in the seated chest press exercise with a neutral grip than in the lying bench press exercise with a grip at 150% of the biacromial width. The PMMP and the PMLP were significantly less activated in the lying bench press exercise with a grip at 50% of the biacromial width compared to the rest of the exercises. The AD muscle activity did not significantly differ in any exercise or grip evaluated. The TB only showed significantly higher activation in the lying bench press exercise with a grip at 50% of the biacromial width than with a grip at 150% of the biacromial width.

Our results agree with previous studies that evaluated muscle activity according to grip width in the horizontal bench press exercise. A study by Barnett et al. (1995) found that the clavicular portion of the pectoralis major and the long head of the TB were more activated with a narrow hand grip (100% of the biacromial distance) than with a wide grip (200% of the biacromial distance). In a subsequent study, Clemons and Aaron (1997) observed that with a narrower grip (100% of the biacromial distance), there was significantly greater muscle activation in the TB than in the pectoralis major. According to those authors, although they did not specify which triceps head they evaluated, the results are justified because the narrower grip may reduce activity towards the end of the lift due to lesser shoulder transverse adduction and perhaps less torque on the shoulder (Clemons and Aaron, 1997). In contrast, Lehman (2005) did not find significant differences in muscle activation when comparing the sternoclavicular and clavicular portions of the pectoralis major between a wide grip (at 200% of the biacromial distance) and a middle grip (at 100% of the biacromial distance) in the bench press exercise. However, that author observed significantly higher activation in the sternoclavicular portion with a wide grip than with a narrow grip (with one hand width distance between the two hands). Likewise, as in the current study, that author also found that TB activity increased when the grip was changed from a wide to a narrower grip (Lehman, 2005). However, in that previous study, the lateral head of the triceps brachii was evaluated rather than the long head.

From the results reported in previous studies and those obtained in the current one, although a significant activation of the TB can be observed with a narrow grip, its muscle activity is the lowest of the four evaluated exercises. However, in our study, the pectoralis major, followed by the AD, was still activated to a greater extent, despite a narrow grip. Nevertheless, Saeterbakken et al. (2017) recommend using a wide grip in a flat bench press exercise if the load is high to improve muscle hypertrophy in athletes.

The seated chest press exercise is a machine-assisted multijoint exercise. Despite limiting the degree of freedom of movement of the joints, in the current study, when evaluating this exercise with a neutral grip (at 150% of the biacromial width), we found slightly greater muscle activity (with no significant differences) in the three portions of the pectoralis major and in the AD, although with a 1RM significantly lower, than in the lying bench press exercise with a grip at 150% of the biacromial distance and the seated chest press exercise with a prone grip. Based on these results, the seated chest press exercise with a neutral grip could be considered a suitable exercise for people who do not need significant strength requirements in their training. A previous study by Balachandran et al. (2016) found similar results with improved physical function in older adults after 12 weeks of strength training in seated machines versus standing cable machines. Similarly, Schott et al. (2019) compared a free-weight training group with a machine-assisted training group and found that after 26 weeks of training, there were similar results on muscular strength in high-functioning older adults.

Nevertheless, following the results obtained in the current study, considering the small influence that occurs when modifying grip widths or the type of exercise (free weights or machine-assisted), the choice of the grip position or exercise should be determined by the athlete's position or the type of movement required for their sport.

Some limitations of the current study should be considered. First, the results were reported in absolute values and were not separated into concentric and eccentric phases. These phases could show differences in muscle activity. However, our purpose was to evaluate muscle activity in the whole movement of the barbell or the machine. Another limitation was our decision to evaluate the movements at a controlled velocity (2 s for concentric and 2 s for eccentric phases). Future studies should evaluate muscle activity at different velocities of execution. Another limitation was that the relative load evaluated was moderate (at 60% 1RM) and selected to improve the participants' security and technique. However, it would have been interesting to evaluate these exercises in a higher load to record the muscle activity in several load resistances. Finally, although normalization to an MVIC was standardized, using procedures previously described in the literature, the MVIC was not matched to the task under investigation. Moreover, grip width was not assessed during the seated chest press exercise because the bar itself assembled on the machine was used. Therefore, there was no possibility of adjusting the grip widths to 150% and 50% of the biacromial width of participants. It would be interesting for future work in which a customized bar could be installed in this machine to adapt the grips to the characteristics of participants.

## Conclusions

Muscle activity of the three portions of the pectoralis major and the AD was similar in the following exercises: lying bench press exercise with a grip at 150% of the biacromial width, seated chest press exercise with a prone grip, and seated chest press exercise with a neutral grip. The lying bench press exercise with a grip at 50% of the biacromial width showed significantly lower muscle activity in the sternal and costal portions of the pectoralis major and greater muscle activity in the TB than during the same exercise with a grip at 150% of the biacromial width. Due to the similar muscle activity found in these exercises, their selection within a strength training program should not be based exclusively on muscle activation, but rather should be justified on the grounds of the load capacity lifted, the level of technique of the participant, and/or the transference to the specific sporting activity.

## Author Contributions

J.M.M. and D.R.-R.; methodology: J.M.M., D.R.-R. and J.M.O.-L.; validation: J.M.M., D.R.-R. and J.M.O.-L.; formal analysis: J.M.M. and D.R.-R.; investigation: J.M.M., D.R.-R. and J.M.O.-L.; resources: J.M.M., D.R.-R. and J.M.O.-L.; data curation: J.M.M. and D.R.-R..; writing—original draft preparation: J.M.M. and D.R.-R.; writing—review & editing: J.M.M., D.R.-R. and J.M.O.-L.; visualization: J.M.M., D.R.-R. and J.M.O.-L.; supervision: J.M.M., D.R.-R. and J.M.O.-L.; project administration: J.M.M.; funding acquisition: J.M.M. All authors have read and agreed to the published version of the manuscript.

## ORCID iD

José M. Muyor: https://orcid.org/0000-0003-2849-0323

José M. Oliva-Lozano: https://orcid.org/0000-0002-7257-3620
